# Supplementary material for: Improving Generalizability of PET DL Algorithms: List-Mode Reconstructions Improve DOTATATE PET Hepatic Lesion Detection Performance
Source: Bioengineering (Basel). 2024 Feb 27;11(3):226. doi: 10.3390/bioengineering11030226 (PMC10968510; doi:10.3390/bioengineering11030226)
Supplement: Supplementary file 1 [file bioengineering-11-00226-s001.zip › bioengineering-2858535-supplementary.pdf]

bioengineering

MDPI

Article

Improving Generalizability of PET DL Algorithms: List-Mode Reconstructions Improve DOTATATE PET Hepatic Lesion Detection Performance

Xinyi Yang [1], Michael Silosky [2], Jonathan Wehrend [3], Daniel V. Litwiller [4], Muthiah Nachiappan [2], Scott D. Metzler [5], Debashis Ghosh [1], Fuyong Xing [1,6,7] and Bennett B. Chin [2,7,*]

1 Department of Biostatistics and Informatics, University of Colorado Anschutz Medical Campus, Aurora, CO, USA; xinyi.2.yang@cuanschutz.edu (X.Y.); debashis.ghosh@cuanschutz.edu (D.G.); fuyong.xing@cuanschutz.edu (F.X.)
2 Department of Radiology, University of Colorado Anschutz Medical Campus, Aurora, CO, USA; michael.silosky@cuanschutz.edu (M.S.); muthiah.nachiappan@cuanschutz.edu (M.N.)
3 Department of Radiology, Santa Clara Valley Medical Center, San Jose, CA, USA; jwehrend@gmail.com
4 GE HealthCare, Denver, CO, USA; daniel.litwiller@ge.com
5 Department of Radiology, University of Pennsylvania, Philadelphia, PA, USA; scott.metzler@uphs.upenn.edu
6 The Computational Bioscience Program, University of Colorado Anschutz Medical Campus, Aurora, CO, USA
7 University of Colorado Cancer Center, University of Colorado Anschutz Medical Campus, Aurora, CO, USA; Fuyong.xing@cuanschutz.edu, bennett.chin@cuanschutz.edu
* Correspondence: bennett.chin@cuanschutz.edu

Supplementary Materials:

A U-Net-like architecture [12] was used for the lesion detection (Figure S1). The neural network has performed well on lesion detection in PET datasets. The down-sampling path contains four residual learning blocks [19]. In the up-sampling path, there are four residual learning blocks and two transposed convolutional layers [20] to aggregate contextual information [21].

The model was optimized with a Combo loss, which is a linear combination of binary cross-entropy loss and Dice loss [22].

$$\mathcal{L}_{COMBO}(y_n, \hat{y}_n) = \alpha\mathcal{L}_{BCE}(y_n, \hat{y}_n) + \mathcal{L}_{DICE}(y_n, \hat{y}_n)$$

$$\mathcal{L}_{BCE}(y_n, \hat{y}_n) = \mathbb{E}_{(x,y)\sim(X,Y)}[-\frac{1}{N}\sum_{n=1}^{N}(\omega\, y_n \log(\hat{y}_n + \varepsilon) + (1 - y_n)\log(1 - \hat{y}_n + \varepsilon))]$$

$$\mathcal{L}_{DICE}(y_n, \hat{y}_n) = \mathbb{E}_{(x,y)\sim(X,Y)}[1 - \frac{\sum_{n=1}^{N} y_n\hat{y}_n + \delta}{\sum_{n=1}^{N} y_n + \hat{y}_n + \delta} - \frac{\sum_{n=1}^{N} b_n\hat{b}_n + \delta}{\sum_{n=1}^{N} b_n + \hat{b}_n + \delta}]$$

where $y_n \in y$ is the ith value of the gold-standard label y and $\hat{y}_n \in \hat{y}$ is the ith value of the corresponding prediction $\hat{y}$.

Implementation Details

We empirically set $\alpha = 6,\ \omega = 5,\ \delta = 1$. We optimize the lesion detection network using stochastic gradient descent with Nesterov momentum: learning rate = 0.0005, momentum = 0.99, batch size = 8, and total iterations = 10^5.

Additionally, a data augmentation module was used to help train the model. The module consists of random shifting, random flipping, and random rotation.

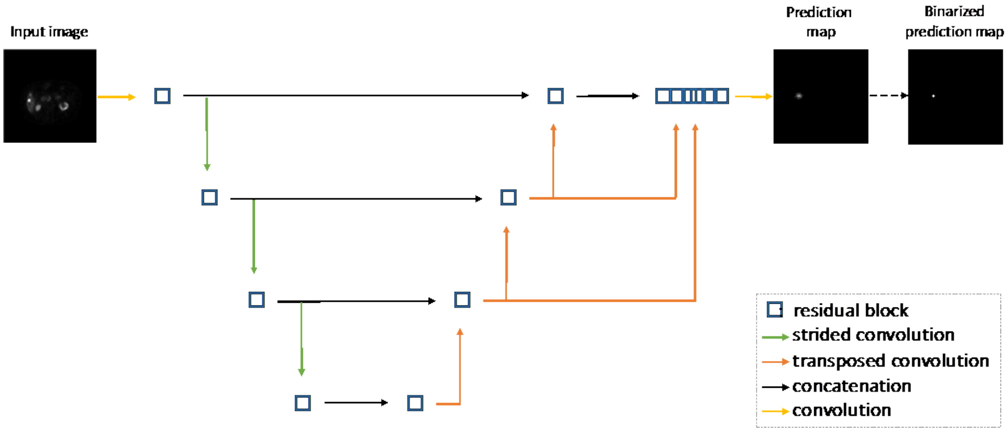

Figure S1. Model Training for Lesion Detection—Network architecture.
